# The quantitative significance of *Syntrophaceae* and syntrophic partnerships in methanogenic degradation of crude oil alkanes

**DOI:** 10.1111/j.1462-2920.2011.02570.x

**Published:** 2011-11

**Authors:** N D Gray, A Sherry, R J Grant, A K Rowan, C R J Hubert, C M Callbeck, C M Aitken, D M Jones, J J Adams, S R Larter, I M Head

**Affiliations:** 1 School of Civil Engineering and Geosciences, Newcastle UniversityNewcastle upon Tyne, NE1 7RU, UK; Departments of 2 Geoscience, University of CalgaryCalgary, Alberta, T2N 1N4, UK.; 3 Biological Sciences, University of CalgaryCalgary, Alberta, T2N 1N4, UK.

## Abstract

Libraries of 16S rRNA genes cloned from methanogenic oil degrading microcosms amended with North Sea crude oil and inoculated with estuarine sediment indicated that bacteria from the genera *Smithella* (*Deltaproteobacteria, Syntrophaceace*) and *Marinobacter* sp. (*Gammaproteobacteria*) were enriched during degradation. Growth yields and doubling times (36 days for both *Smithella* and *Marinobacter*) were determined using qPCR and quantitative data on alkanes, which were the predominant hydrocarbons degraded. The growth yield of the *Smithella* sp. [0.020 g_(cell-C)_/g_(alkane-C)_], assuming it utilized all alkanes removed was consistent with yields of bacteria that degrade hydrocarbons and other organic compounds in methanogenic consortia. Over 450 days of incubation predominance and exponential growth of *Smithella* was coincident with alkane removal and exponential accumulation of methane. This growth is consistent with *Smithella's* occurrence in near surface anoxic hydrocarbon degrading systems and their complete oxidation of crude oil alkanes to acetate and/or hydrogen in syntrophic partnership with methanogens in such systems. The calculated growth yield of the *Marinobacter* sp., assuming it grew on alkanes, was [0.0005 g_(cell-C)_/g_(alkane-C)_] suggesting that it played a minor role in alkane degradation. The dominant methanogens were hydrogenotrophs (*Methanocalculus* spp. from the *Methanomicrobiales*). Enrichment of hydrogen-oxidizing methanogens relative to acetoclastic methanogens was consistent with syntrophic acetate oxidation measured in methanogenic crude oil degrading enrichment cultures. qPCR of the *Methanomicrobiales* indicated growth characteristics consistent with measured rates of methane production and growth in partnership with *Smithella*.

## Introduction

Methanogenic degradation of pure hydrocarbons and hydrocarbons in crude oil proceeds with stoichiometric conversion of individual hydrocarbons to methane and CO_2_. (Zengler *et al*., 1999; Anderson and Lovley, 2000; Townsend *et al*., 2003; Siddique *et al*., 2006; Gieg *et al*., 2008; 2010; Jones *et al*., 2008; Wang *et al*., 2011). Comparison of methanogenic degradation of crude oil in estuarine sediment microcosms with patterns of hydrocarbon removal in degraded petroleum reservoirs has suggested that preferential removal of alkanes in biodegraded petroleum reservoirs is driven by methanogenesis and is probably responsible for the formation of the world's deposits of heavy oil (Jones *et al*., 2008). It has even been proposed that stimulation of *in situ* methanogenic biodegradation of crude oil may be harnessed to enhance energy recovery from petroleum reservoirs (Gieg *et al*., 2008; Jones *et al*., 2008; Gray *et al*., 2009; Mbadinga *et al*., 2011). From a wider perspective hydrocarbons are common contaminants of surface and shallow environments (Keith and Telliard, 1979; Hippensteel, 1997) and *in situ* methanogenic biodegradation of crude oil is an important component of the attenuation of contaminant plumes in such environments (Gray *et al*., 2010).

A meta-analysis of microbial communities in hydrocarbon impacted environments has indicated that communities in near surface sediments are distinct from those found in deeper warmer petroleum reservoirs (Gray *et al*., 2010; Wang *et al*., 2011). On this basis the estuarine system studied by Jones and colleagues (2008) best represents a model for direct comparison with near surface hydrocarbon impacted environments contaminated with crude oil. In comparison with pure hydrocarbons, crudeoils contain a complex mixture of chemicals including refractory and or toxic components in addition to degradable hydrocarbons. This complexity is likely to influence the activity and selection of alkane degrading microorganisms enriched on crude oil. Here we have determined the relative importance of different organisms potentially involved in methanogenic crude oil degradation in surface sediments by quantification of their growth in relation to methane production and removal of crude oil alkanes.

## Results

### Methanogenic oil degradation

**Fig. 1:**
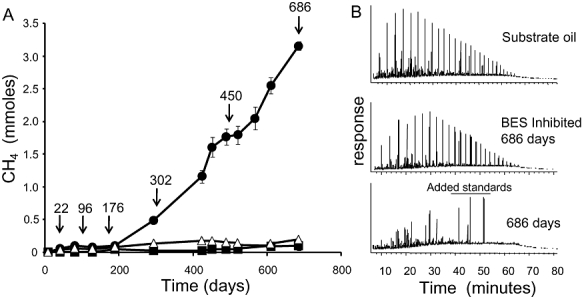
A. Methane production from 300 mg North Sea crude oil added to estuarine sediments incubated under methanogenic conditions in laboratory microcosms (100 ml) for 686 days. The error bars show ±1 × standard error (*n* = 3). Closed circles indicate methane production from crude oil amended microcosms and open triangles indicate methane production from microcosms, which did not receive crude oil (data previously presented in Jones *et al*., 2008). Closed squares indicate methane production from crude oil and BES amended microcosms. Arrows indicate sacrificial sampling events at 22, 176, 302, 450 and 686 days. B. Gas chromatograms of total hydrocarbon fractions from the undegraded substrate oil, crude oil amended and crude oil and BES amended microcosms incubated for 686 days. Time 0 on the chromatograms, which are displayed from 5 to 80 min, corresponds to injection.

Oil conversion to methane in oil degrading microcosms inoculated with estuarine sediment and amended with North Sea oil was confirmed by comparison of methane yields and oil alterations in oil amended, unamended and (BES) inhibited control microcosms (Fig. 1). Methanogenic oil degradation was characterized by an apparent 200 day lag phase (Fig. 1A). Methane production correlated stoichiometrically with the removal of alkanes (*n*C_7_-*n*C_34_) (Jones *et al*., 2008, Fig. 1B). Furthermore, near complete degradation of alkanes occurred before any significant removal of aromatic hydrocarbon (Jones *et al*., 2008). Microcosms sacrificially sampled after 0, 22, 176, 302, 450 and 686 days for analysis of residual crude oil were also used for analysis of the microbial communities present.

### Denaturing gradient gel electrophoresis (DGGE) analysis of bacterial and archaeal communities

Denaturing gradient gel electrophoresis analysis of the microbial communities in replicate microcosms over time demonstrated reproducible changes associated with degradation of the crude oil and methane formation relative to control microcosms with no oil added. There was a high degree of similarity in the DGGE profiles of communities from replicate microcosms that were sacrificially sampled at different time points (Table 1). On day 22 there was no significant difference in the bacterial or archaeal community profiles between treatments; whereas, at 302 and 686 days there were statistically significant differences between treatments in both the bacterial and archaeal communities (Table 1, Fig. S1).

**Table 1:** Average similarity between bacterial and archaeal DGGE profiles from methanogenic microcosms

	Bacterial profiles		Archaeal profiles	
		
Time (days)	Similarity within replicates	Similarity between oil and no-oil treatments	Similarity within replicates	Similarity between oil and no-oil treatments
22	84.7 ± 5.1	79.4 ± 4.5	80.8 ± 5.0	75.1 ± 4.1
302	79.1 ± 7.2*	32.7 ± 4.7*	73.3 ± 3.4*	57.3 ± 1.1*
686	72.8 ± 1.6*	36.9 ± 5.8*	81.1 ± 3.8*	40.3 ± 3.8*

The asterisk indicates a statistically significant difference between oil treated and untreated samples (*t*-test *P* < 0.05).

### Bacterial and archaeal community composition in Tyne sediment inoculum

**Fig. 2:**
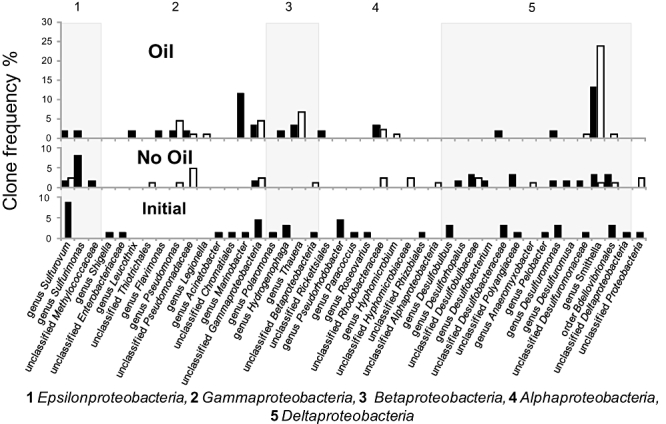
Phylogenetic affiliation of *proteobacterial* 16S rRNA sequences recovered from methanogenic microcosms. Clone frequency in bacterial 16S rRNA gene clone libraries from the inoculum and initial day 22 samples (bottom panel, total number of clones = 71) and in samples from methanogenic oil degrading microcosms (top panel, number of ‘day 302’ clones = 61, number of ‘day 686’ clones = 87) and control microcosms with no added oil (middle panel, number of ‘day 302’ clones = 62, number of ‘day 686’ clones = 87). Data from clone libraries from day 302 (filled bars) and day 686 (open bars) are shown. Clones were grouped into categories based on their genus, order, class or phylum level affiliation after phylogenetic analysis with the ARB software package using an RDP guide tree. The affiliation of individual sequences was cross-checked using the RDP taxonomical hierarchy with the Naïve Bayesian rRNA Classifier Version 2.0, July 2007.

**Fig. 3:**
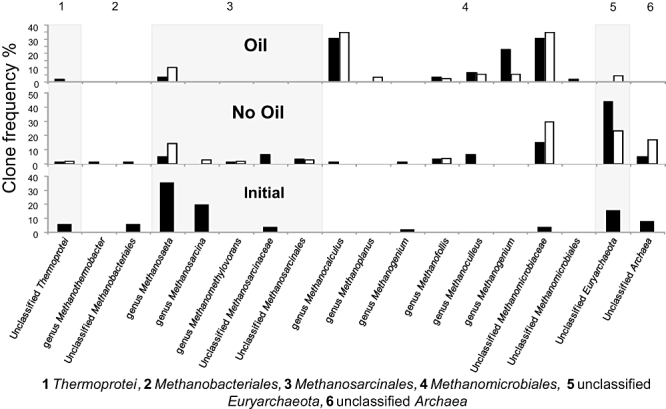
Phylogenetic affiliation of archaeal 16S rRNA sequences recovered from methanogenic microcosms. Clone frequency distributions in archaeal 16S rRNA gene clone libraries from the inoculum and initial day 22 samples (bottom panel) and in samples from the methanogenic oil degrading microcosms (top panel) and control microcosms with no added oil (middle panel). Data from clone libraries from day 302 (filled bars) and day 686 (open bars) are shown. Clones were grouped into categories based on their genus, order, class or phylum level affiliation after phylogenetic analysis with the ARB software package using an RDP guide tree. The affiliation of individual sequence was cross-checked using the RDP Taxonomical hierarchy with the Naive Bayesian rRNA Classifier Version 2.0, July 2007.

**Fig. 5:**
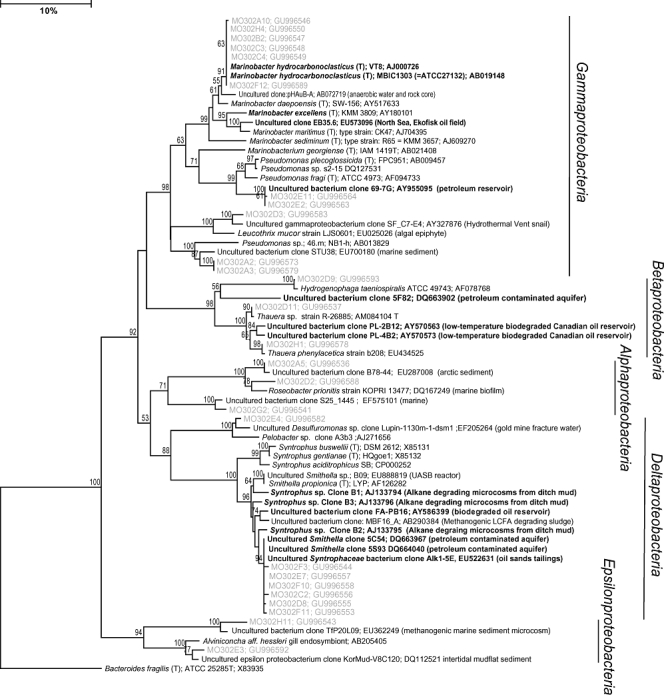
Phylogenetic distance trees based on comparative analysis of Proteobacterial partial 16S rRNA sequences recovered from a representative oil amended microcosm on day 302. Sequences recovered in this study (grey text) are prefixed by MO302. Related organisms identified in petroleum systems or those directly implicated in oil degradation are in bold. GenBank accession numbers for all database sequences are provided in parenthesis. Tree rooted with respect to the *Bacteroides fragilis* ATCC 25285^T^, 16S rRNA sequence (X83935). The scale bar denotes 10% sequence divergence and the values at the nodes indicate the percentage of bootstrap trees that contained the cluster to the right of the node. Bootstrap values less than 50 are not shown.

**Fig. 7:**
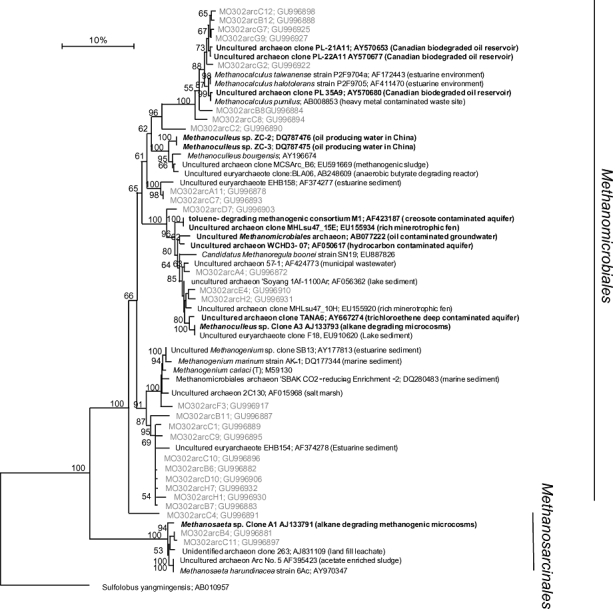
Phylogenetic distance tree based on the comparative analysis of archaeal partial 16S rRNA sequences recovered from a representative oil amended microcosm on day 302. Sequences recovered in this study (grey text) are prefixed by MO302. Related organisms identified in petroleum systems or those directly implicated in oil degradation are in bold. GenBank accession numbers for all database sequences are provided in parenthesis. Tree rooted with respect to the *Sulfolobus yangmingensis* 16S rRNA sequence (AB010957). The scale bar denotes 10% sequence divergence and the values at the nodes indicate the percentage of bootstrap trees that contained the cluster to the right of the node. Bootstrap values less than 50 are not shown.

More detailed bacterial community analysis was conducted on microcosm samples from day 22, 302 and 686 using 16S rRNA gene clone libraries (see Fig. 2 legend for individual library sizes). Clone libraries for individual samples were prepared on the basis that there was high similarity between DGGE profiles from replicate samples and therefore individual samples were considered to be representative. 16S rRNA sequences from the sediment microcosms were classified using the RDP Naïve Bayesian rRNA Classifier (Wang *et al*., 2007). Samples taken on day 22 and from the sediment inoculum were compared using the RDP library compare tool and were found not to be significantly different for the bacterial or archaeal genera (*P* > 0.05). The 16S rRNA sequence data from these samples were considered together and are referred to as the initial community [Fig. 2 (Proteobacteria), Fig. 3. (Archaea), Fig. S2 (all other bacterial taxa)]. The closest matches to the 16S rRNA sequences in the EMBL database were recovered from lake, estuarine, marine, mangrove and cold seep sediments as well as sequences recovered from anaerobic sludges and soil (data not shown). A number of these bacterial or archaeal sequences were most closely related to organisms previously identified in polluted sediments including those contaminated with petroleum hydrocarbons (*c.* 14%, accession numbers GU996598, GU996609, GU996619, GU996620, GU996621, GU996627, GU996629, GU996634, GU996635, GU996636, GU996659, GU996660, GU996663, GU996690, GU996691, GU996693, GU996712 in Figs 5 and 7). These data are consistent with the long industrial history of the river Tyne, which was the first major coal exporting port to develop during the industrial revolution. The most frequently recovered archaeal sequences were from the genus *Methanosaeta* (35.3% of clones) and the genus *Methanosarcina* (19.6% of clones) both from the order *Methanosarcinales* (Fig. 3, bottom panel). *Methanomicrobiales* and *Methanobacteriales* together comprised only 11.7% of clones.

### Structure and dynamics of bacterial communities during methanogenic crude oil biodegradation

**Fig. 4:**
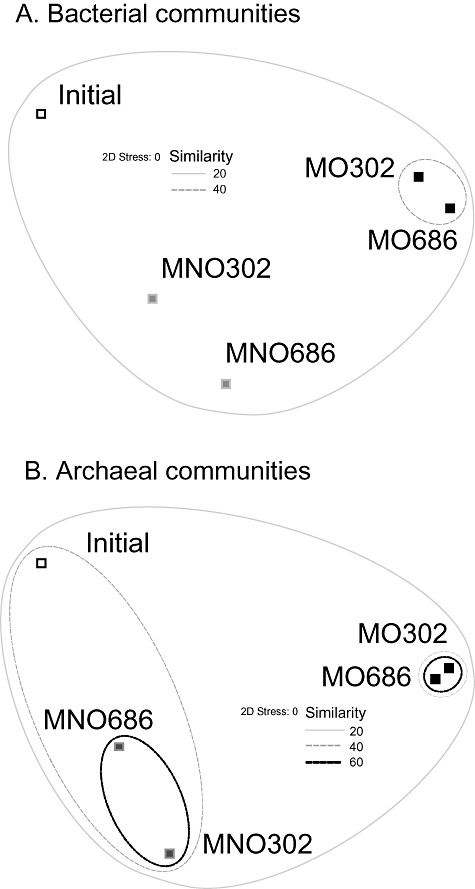
Non-metric MDS analysis of the bacterial and archaeal communities present in the River Tyne sediment at the start of the incubation period (Initial) and after 302 and 686 days based on OTU frequencies in 16S rRNA gene clone libraries. MDS plots are representations of how different the communities are from each other based on clustering of like samples. Similarity contour lines from cluster analyses are superimposed on to the MDS plots; however, only contours encompassing more than one clone library profile are shown. The MO302 and MO686 symbols indicate the oil amended microcosms and MNO302 and MNO686 symbols indicate the unamended microcosms.

Consistent with the DGGE analysis of replicate samples, sequences from methanogenic oil degrading microcosms incubated for 302 and 686 days indicated major changes in the bacterial communities compared with the initial and unamended microcosm communities [Fig. 2 (Proteobacteria) and Fig. S2 (all other bacterial taxa)]. Ordination of 16S rRNA gene clone frequency data by non-metric multidimensional scaling (MDS) indicated that methanogenic oil degrading communities from independent microcosms, sampled on day 302 and day 686 clustered together (Fig. 4A). The similarity of the day 302 and 686 communities (which were obtained from separate, sacrificially sampled microcosms) was also observed in DGGE analysis of replicate samples and supports the notion that the clone library data are genuinely representative. The SIMPER routine in PRIMER 6 (Clarke and Warwick, 2001) was used to determine the contribution of different operational taxonomic units (OTUs) to the average similarity of oil-treated microcosms. This discrimination tool identified the deltaproteobacterial genus *Smithella* as the largest contributor (16%) to the overall similarity of the oil amended communities. The representation of this taxon was significantly greater in clone libraries from the oil degrading microcosms on day 302 and 686 compared with the initial community where it was not detected (RDP library compare tool *P* = 0.002 and < 0.001 respectively) and compared with unamended control microcosms sampled on day 302 and 686 (*P* = 0.004 and < 0.001). Sequences from the gammaproteobacterial genus *Marinobacter* were significantly enriched in oil-treated microcosms on day 302 relative to the initial community and unamended controls (RDP library compare tool *P* = 0.004 and 0.007). However, *Marinobacter* sequences were no longer detected in samples from day 686. In addition to *Smithella* and *Marinobacter,* other taxa, notably *Thauera* (Proteobacteria) and *Anaerolinea* (Chloroflexi), were enriched but to a lesser extent in the methanogenic oil degrading microcosms (Fig. 2 (Proteobacteria) and Fig. S2 (all other bacterial taxa)). Many of the sequences recovered at high frequency in clone libraries from methanogenic oil degrading microcosms, including *Smithella* and *Marinobacter,* were found to be most closely related to organisms directly implicated in petroleum degradation [Fig. 5 (Proteobacteria) and Fig. S3 (all other bacterial taxa)].

**Fig. 6:**
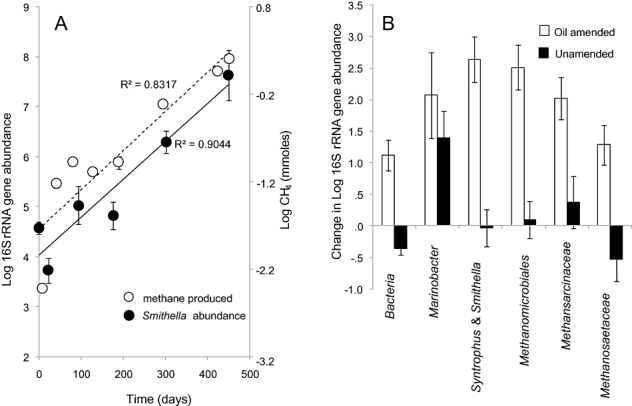
A. *Smithella*/*Syntrophus* 16S rRNA gene abundances (log gene abundance/cm^3^) (closed circles) and log methane produced (open circles) in the oil degrading microcosms from 0 to 450 days. B. Average differences in 16S rRNA gene abundance (log gene abundance/cm^3^) between the lag phase (0–176 days) and the period of highest methane production (176–450 days) for the taxonomic groups targeted by qPCR assays. A value of zero indicates no change, a positive value indicates an increase in abundance and a negative value a decrease. Error bars represent 1 × SE.

On the basis of increased representation in 16S rRNA gene clone libraries a qPCR analysis targeting *Smithella* and related *Syntrophus* spp. within the family *Syntrophaceae* was used to determine 16S rRNA gene abundances in triplicate samples taken on day 22, 94, 176, 302, 450 and 686. The qPCR data for *Smithella/Syntrophus* gave an excellent fit (R^2^ = 0.9) to an exponential growth model up to 450 days (Fig. 6A). In this model (*N_T_* = *N_0_*e*^µt^*) *N_T_* is the abundance of *Smithella/Syntrophus* at time *T, N_0_* is the abundance of *Smithella/Syntrophus* at time *T_0_. t* is time in days and *µ* is the specific growth rate (day^−1^). Exponential growth coincided with the exponential increase in methane produced during the same time period (Fig. 6A). The average increase in abundance of *Smithella/Syntrophus* between the early phase of hydrocarbon degradation (0–176 days) and the following period (176–450 days) was 2.64 ± 0.41 log units [Figs 6B, *P* < 0.001; two-tailed *t*-test (assuming unequal variance)]. Changes in abundance were not significant over the same time period in the unamended microcosms (Fig. 6B). The specific growth rate, doubling time and growth yield on crude oil alkanes for *Smithella/Syntrophus* were calculated between day 0 and 450 days. The specific growth rate (*µ*) was 0.019 day^−1^ corresponding to a doubling time of 36 days and the growth yield was 0.020 g_(cell-C)_/g_(alkane-C)_ assuming that it was responsible for all alkane degradation.

qPCR data for *Marinobacter* gave a weaker fit (R^2^ = 0.61) to an exponential growth model between day 0 and 450 days (data not shown). Log gene abundance of the *Marinobacter* spp. targeted by the qPCR assay increased by 2.1 ± 0.68 log units in the oil amended microcosms ((*P* = 0.021, two-tailed *t*-test assuming unequal variance). In contrast to *Smithella/Syntrophus, Marinobacter* gene abundance also increased in the unamended microcosms by 0.78 ± 0.31 log units (*P* = 0.02, two-tailed *t*-test assuming unequal variance). The specific growth rate (*µ*) calculated for *Marinobacter* sp. in the amended microcosms was similar to that determined for *Smithella*/*Syntrophus* (0.019 day^−1^; doubling time 36 days). However, the initial log of gene abundance (copies/cm^3^) of *Marinobacter* (3.25 ± 0.29) was more than an order of magnitude lower than *Smithella/Syntrophus* (4.57 ± 0.13) and the estimated growthyields for *Marinobacter* was only 0.0005 g_(cell-C)_/g_(alkane-C)_ if one assumes that it was responsible for all alkane degradation.

### The structure and dynamics of archaeal communities during methanogenic crude oil biodegradation

Major changes in archaeal communities were noted in methanogenic oil degrading microcosms, specifically there was a significant increase in *Methanomicrobiales*-related sequences relative to the initial community and all unamended control microcosms (*P* < 0.001 RDP library compare tool; Fig. 3). Ordination of OTU frequency data by MDS showed the same pattern of separation based on oil treatment as observed for the bacteria (Fig. 4). SIMPER analysis demonstrated that *Methanocalculus* sequences and unclassified *Methanomicrobiaceae* each contributed 29% towards the similarity of the oil degrading libraries. For all clone library comparisons the enrichment of *Methanocalculus* was highly significant in the oil degrading microcosms (*P* < 0.001; RDP library compare tool). Phylogenetic analysis of the archaeal sequences recovered from the oil degrading microcosms (Fig. 7) showed that many were closely related to organisms identified in petroleum systems or those directly implicated in oil degradation. Microcosms that were not treated with crude oil were dominated by unclassified *Euryarchaeota*, unclassified *Archaea*, unclassified *Methanomicrobiaceae* and *Methanosaeta* sequences (Fig. 3).

Archaeal gene abundances increased significantly in the oil amended microcosms between 0–176 days and 176–450 days. For instance, the log gene abundance of *Methanomicrobiales* increased by 2.51 ± 0.54 log units in the oil amended microcosms (*P* = 0.004; two-tailed *t*-test assuming unequal variance); *Methanosarcinaceae* increased by 2.0 ± 0.33 log units (*P* < 0.001) and *Methanosaetaceae* increased by 1.29 ± 0.31 log units (*P* = 0.03). Changes in archaeal abundance in the unamended microcosms were not significant.

The degree of enrichment of *Methanomicrobiales* was not significantly different to that observed for *Methanosarcinaceae* and was therefore less than might have been expected given the relative increase in the frequency of *Methanomicrobiales* sequences observed in 16S rRNA gene clone libraries from day 302 and 686 (Fig. 3). The qPCR data for the different methanogen groups were a good fit to an exponential growth model between 0 and 450 days (data not shown) with R^2^ values of 0.92, 0.78 and 0.73 for the *Methanomicrobiales, Methanosarcinaceae* and *Methanosaetaceae* respectively. The respective growth yields (assuming the contribution of individual methanogen groups to methane production from alkane removal was proportionate to their relative abundances) were 0.009–0.036, 2–2.2 × 10^−3^ and 3.0–3.5 × 10^−6^ g_(cell-C)_/g_(alkane-C)_ (see experimental procedures for explanation of the range of values). The total methanogen growth yield calculated from the combined growth of all three methanogen groups on alkane removed was 0.011–0.038 g_(cell-C)_/g_(alkane-C)_. Expressed in terms of moles of methane produced the individual growth yields were 0.141–0.565, 0.031–0.035, 4.6–5.5 × 10^−5^ g_(cell-C)_/mol _(CH4)_ and the total methanogen growth yield was 0.17–0.6 g_(cell-C)_/mol _(CH4)_. Calculated specific growth rates were 0.02, 0.01 and 0.01 day^−1^ respectively. Doubling times for *Methanomicrobiales* were 34 days whereas the *Methanosarcinaceae* and *Methanosaetaceae* required 69 days.

### Pathways of acetate-stimulated methanogenesis in methanogenic oil degrading microcosms

**Fig. 8:**
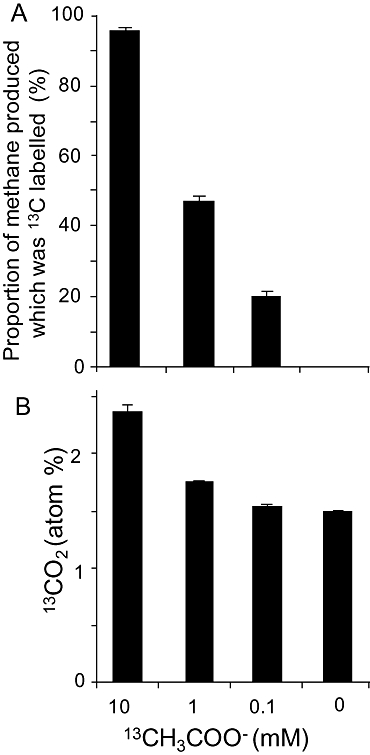
(A) Proportion of ^13^CH_4_ and ^12^CH_4_ and (B) proportion of ^13^CO_2_ (atom %) in the headspace of methanogenic oil degrading enrichments amended with different concentrations of 2-^13^C sodium acetate (10, 1, 0.1 mM). Error bars represent 1 × SE.

To determine the fate of acetate during methanogenesis subsamples from the methanogenic oil degrading microcosms were treated with 2-^13^C sodium acetate and production of ^13^CO_2_, ^13^CH_4_ and ^12^CH_4_ were compared with unamended controls. Maximum total methane production rates from acetate were 33.8 ± 0.36 nmoles h^−1^, 2.6 ± 0.2 nmoles h^−1^, 2.3 ± 0.1 nmoles h^−1^, 0.06 ± 0.01 nmoles h^−1^ in experiments to which 10, 1, 0.1 or 0 mM 2-^13^C sodium acetate were added. Theoretically the direct cleavage of 99 atom% 2-^13^C acetate to methane and CO_2_ by acetoclastic methanogens (Table 2, Eq. 1) should produce 100% ^13^CH_4_ with no production of ^13^CO_2_. However, if the principal sink for acetate was syntrophic actetate oxidation (Table 2, Eq. 2) coupled to hydrogenotrophic methanogenesis (Table 2, Eq. 3) a lower proportion of ^13^CH_4_ methane would be produced because the ^13^CO_2_ from syntrophic acetate oxidation (SAO) would be diluted with ^12^C from the carbonate buffered medium, before reduction of CO_2_ to methane. The proportion of ^13^CH_4_ from ^13^CH_3_COO^-^ was lower than 100% (Fig. 8A) indicating that SAO was occurring in the methanogenic oil degrading system. In addition to the production of unlabelled methane, SAO was confirmed by the formation of ^13^CO_2_ above natural abundance from oxidation of C-2 of the labelled acetate to ^13^CO*_2_* (Fig. 8B).

**Table 2:** Reactions involved in the methanogenic degradation of alkanes (hexadecane)

Process	Reaction	Eq.
Acetoclastic methanogenesis	CH_3_COO^-^ + H^+^→ CH_4_ + CO_2_	1
Syntrophic acetate oxidation (SAO)	CH_3_COO^-^ + H^+^ + 2H_2_O → 4H_2_ + CO_2_	2
Hydrogenotrophic methanogenesis	4H_2_ + CO_2_→ CH_4_ + 2H_2_O	3
Syntrophic alkane oxidation to acetate and hydrogen	4C_16_H_34_ + 64H_2_O → 32CH_3_COO^-^ + 32H^+^ + 68H_2_	4
Alkane degradation to methane	4C_16_H_34_ + 30H_2_O → 15CO_2_ + 49CH_4_	5

The proportion of ^13^CH_4_ production compared with total CH_4_ production was dependent on the initial concentration of acetate (Fig. 8A). With 0.1 mM ^13^CH_3_COO^-^, 20% of the methane produced was labelled indicating acetoclastic methanogenesis was a minor component of biogenic methane production; however, with 1 mM ^13^CH_3_COO^-^, approximately 50% of the methane produced was labelled and with 10 mM ^13^CH_3_COO^-^, most of the methane produced was ^13^C-labelled (Fig. 8A). Despite the high proportion of labelled methane production in experiments amended with 10 mM ^13^CH_3_COO^-^ and the likely dominance of acetoclastic methanogenesis in this system, there was a statistically significant production of ^13^CO*_2_* above that observed in the unamended controls (Fig. 8B, *P* < 0.001, *t*-Test). This was also true of experiments with 1 mM ^13^CH_3_COO^-^ (*P* = 0.014). However, despite the lower proportion of labelled methane found in the experiments amended with 0.1 mM acetate, indicating SAO, incorporation of label into CO_2_ was not significantly different from the unamended controls (*P* > 0.05). This is most likely explained by the absolute amounts of ^13^CH_3_COO^-^ present that differed by three orders of magnitude and thus the low absolute ^13^CO_2_ yield relative to the background dissolved inorganic carbon pool in the carbonate buffered nutrient medium (30 mM).

## Discussion

During the methanogenic degradation of crude oil, alkanes are oxidized syntrophically to methanogenic substrates (Table 2, Eq. 4), which are in turn converted to methane and CO_2_ (Table 2, Eqs 1, 2 and 3). The overall reaction for the degradation of *n*-alkanes (e.g. hexadecane, Table 2, Eq. 5) in oil amended microcosms was confirmed by generation of stoichiometric amounts of methane (Jones *et al*., 2008). Syntrophic oxidation of acetate to H_2_ and CO_2_ (Table 2, Eq. 2) during conversion of alkanes in crude oil to methane, has been suggested as an alternative to acetoclastic methanogenesis (Table 2, Eq. 1) and Rayleigh fractionation modelling has indicated that within petroleum reservoirs, a large proportion of the acetate generated from hydrocarbon degradation can be channelled through SAO (Jones *et al*., 2008).

### The role of *Syntrophaceae* in low temperature hydrocarbon degrading systems

16S rRNA gene clone libraries and qPCR indicated an important role for bacteria related to the genera *Smithella* and *Syntrophus* in methanogenic crude oil degrading consortia. The relationship between *Smithella* and *Syntrophus* is at present unclear with 16S rRNA sequences from uncultured bacteria designated as *Syntrophus* sp. clustering with those named as *Smithella* (Fig. 5). However, our phylogenetic analysis provides strong bootstrap support (100%) for the separation of *Smithella propionica* and related sequences from uncultured organisms (including those enriched in this study) from cultured *Syntrophus* spp. On the basis of this analysis the *Syntrophaceae* sequences (e.g. MO302D8) enriched in the methanogenic oil degrading microcosms are considered to represent *Smithella* spp. Furthermore, we propose that the sequences designated as *Syntrophus* (Clones B1, B2 and B3) from a methanogenic hexadecane degrading enrichment (Zengler *et al*., 1999) also represent *Smithella* sp.. These organisms were named as *Syntrophus* sp. because the genus *Smithella* was not described at the time of Zengler and colleagues work (Liu *et al*., 1999).

A number of strands of evidence from this study and the wider literature provide collective evidence for a direct role for *Syntrophaceae* in the activation and oxidation of crude oil alkanes via long chain fatty acids (LCFA) to acetate and hydrogen in methanogenic environments. First, the calculated growth yield [0.02 g_(cell-C)_/g_(alkane-C)_] for the enriched *Smithella* species when added to the maximum methanogen growth yield [0.038 g_(cell-C)_/g_(alkane-C)_] is consistent [0.058 g_(cell-C)_/g_(alkane-C)_] with a quantitatively important role for *Syntrophaceace* in the degradation of alkanes in the oil degrading microcosms. For comparison, growth yields for methanogenic consortia growing on various organic compounds range from 0.02 to 0.1 g_(cell-C)_/g_(substrate-C)_. [values from the literature were recalculated in terms of g_(cell-C)_/g_(substrate-C)_ to allow direct comparison with our results]. These substrates include formate (0.02, Dolfing *et al*., 2008a), benzoate (0.038, Auburger and Winter, 1995), lactate (0.055, Walker *et al*., 2009), glycerol (0.08, Qatibi *et al*., 1998), pyruvate (0.09, Walker *et al*., 2009), xylene (0.09, Edwards and Grbić-Galić, 1994), Toluene (0.1, Edwards and Grbić-Galić, 1994). With respect to the thermodynamics of alkane degradation in the crude oil degrading microcosms the combined growth yield should be most comparable with those found for toluene and xylene. When normalized for the number of carbon atoms, these compounds have free energy yields [toluene, −18.7 KJ mol^−1^ carbon and xylene −21.1 KJ mol^−1^ carbon (Edwards and Grbić-Galić, 1994)], which are similar to those calculated for alkanes (Dolfing *et al*., 2008b) i.e. C8-C80 alkanes (−23.1 to –23.4 KJ mol^−1^ carbon). The small discrepancy between the growth yields we have calculated and those for methanogenic degradation of toluene or xylene may be explained by our assumption in growth yield calculations that *Smithella* was responsible for the degradation of all alkanes to acetate and H_2_. In reality, the oil degrading microcosms contained a range of bacteria some of which may have utilized a proportion of the alkanes degraded.

Another strand of evidence implicating *Smithella* in methanogenic crude oil degradation is the large number of studies that have identified *Syntrophaceae* as dominant organisms in hydrocarbon impacted systems (e.g. Dojka *et al*., 1998; Zengler *et al*., 1999; Bakermans and Madsen, 2002; Kasai *et al*., 2005; Allen *et al*., 2007; Shimizu *et al*., 2007; Ramos-Padrón *et al*., 2011; Figs 2 and 5; Table 3). With the exception of the study of Shimizu and colleagues (2007), all these studies were of near surface soils, sediments, oil tailings ponds or aquifers. Critically, the association of a specific subgroup of the genus *Smithella* (Fig. 5) with anaerobic hydrocarbon environments suggests that these *Smithella* may be directly involved in the degradation of the hydrocarbons present. *S. propionica,* the only *Smithella* sp. in pure culture, is not known to degrade LCFA but in common with many other syntrophic bacteria it does degrade compounds such as acetate, propionate or butyrate. These short chain fatty acids are ubiquitous intermediates in organic matter degradation in all anoxic environments and as such these substrates are less likely to be drivers for the selection of one specific group of syntrophic bacteria. The consistent association of *Smithella* spp. with hydrocarbon impacted environments therefore implies that they are not selected simply by short chain fatty acids produced from alkane oxidation, but rather specifically because they have the ability to degrade hydrocarbons.

**Table 3:** A survey of oil and hydrocarbon associated *Syntrophaceae*.^a^

Study reference	Clone/strain	Accession	Source environment	Region	%^b^
This study	MO302D8	GU996555	Methanogenic hydrocarbon degrading enrichment	UK	100
Penner *et al*. (Genbank)^c^	Alk1-5E	EU522631	Methanogenically degrading oil sands tailings	Canada	99
Allen *et al*. (2007)	Clone 5C54	DQ663967	Hydrocarbon contaminated sediments	Canada	99
Zengler *et al*. (1999)	Clone B2	AJ133795	Methanogenic hexadecane degrading consortium (ditch mud)	Germany	98
Hatamoto *et al*. (2007)	Clone : MBF16_A	AB290384	Methanogenic LCFA degrading sludge	Japan	97
Grabowski *et al*. (2005)	FA-PB16	AY586399	Oil field production water degrading LCFAs methanogenically	Canada	94
Shimizu *et al*. (2007)	YWB12	AB294281	Methanogenic Coal seam groundwater	Japan	94
Shimizu *et al*. (2007)	YWB13	AB294282	Methanogenic Coal seam groundwater	Japan	94
She and Zhang (Genbank)^c^	DQ315-22	EU050698	Oil field production water	China	93
Zengler *et al*. (1999)	B1	AJ133794	Methanogenic hexadecane degrading consortium	Germany	93
Dojka *et al*. (1998)	WCHB1-12	AF050534	hydrocarbon contaminated aquifer	USA	93
Penner *et al*. (Genbank)^c^	Alk2-2B	EU522633	Degrading oil sands tailings	Canada	93
Penner *et al*. (Genbank)^c^	MLSB_6 m_11C_B	EF420213	Degrading oil sands tailings	Canada	93
Zengler *et al*. (1999)	B3	AJ133796	Methanogenic hexadecane degrading consortium	Germany	93
Grabowski *et al*. (2005)	FA-PB5	AY586395	Oil field production water	Canada	92
Penner *et al*. (Genbank)^c^	BTEX1-10B	EU522637	Oil sands tailings enrichment culture	Canada	92
Penner *et al*. (Genbank)^c^	Nap2-2C	EU522636	Oil sands tailings enrichment culture	Canada	91
Jackson *et al*. (1999)	*S. aciditrophicus*	U86447	Sewage treatment plant	USA	91
Penner *et al*. (Genbank)^c^	LCA1-1C	EU522632	Oil sands tailings enrichment culture	Canada	91
Bakermans and Madsen (2002)	12–2	AF351212	Coal-tar-waste-contaminated aquifer	USA	90
Bakermans and Madsen (2002)	8–45	AF351238	Coal-tar-waste-contaminated aquifer	USA	90
Bakermans and Madsen (2002)	36–11	AF351220	Coal-tar-waste-contaminated aquifer	USA	90
Brofft *et al*. (2002)	FW99	AF523966	Coal impacted wetland	USA	90
Gieg *et al*. (2008)	lg1d02	EU037972	Gas condensate-contaminated aquifer	USA	89
Orcutt *et al*. (Genbank)^c^	GoM161_Bac20	AM745136	Oil impacted sediments	Marine	89
Winderl *et al*. (2008)	D25_29	EU266903	Hydrocarbon contaminated aquifers	Germany	89
Winderl *et al*. (2008)	D25_39	EU266912	Hydrocarbon contaminated aquifers	Germany	89
She and Zhang (Genbank)^c^	DQ315-4	EU050697	Oil field production water	China	89
Kasai *et al*. (2005)	clone:BSC50	AB161292	Hydrocarbon contaminated soils	Japan	87
Chauhan and Ogram (2006)	F1A25	DQ201587	Acetate utilizing microorganisms in soil	USA	87

a Family level assignment based on RDP taxonomic classification.

b % sequence identity with the MO302D8 (this study) determined by BLAST (Altschul *et al*., 1990).

c No accompanying journal publication.

Additional support for enrichment of the *Smithella* on crude oil alkanes comes from the known physiology of close relatives. Although, the only cultured *Smithella* sp., *S. propionica* is a propionate oxidizer (Liu *et al*., 1999), organisms with higher 16S rRNA sequence identity to MO302D8 have been implicated in the degradation of LCFA. A member of the *Syntrophaceae* related to *Smithella* spp., which shared 97% 16S rRNA sequence identity with MO302D8 from the current study, was isotopically enriched in a methanogenic sludge amended with ^13^C labelled palmitate (Fig. 5, AB290384; Hatamoto *et al*., 2007). Grabowski and colleagues (2005) also enriched (90–100% of the bacterial population) a member of the *Syntrophaceae* (clone FA-PB16; AY586399 in Fig. 5 and 94% 16S rRNA sequence identity with MO302D8) from a low temperature, shallow, oil field production water on LCFA (heptadecanoate and stearate).

There is thus precedence for organisms related to those enriched in the methanogenic crude oil degrading microcosms being capable of LCFA oxidation. Are these same organisms likely responsible for conversion of alkanes to LCFA, the key to alkane degradation? In our crude oil amended methanogenic microcosms or indeed any anaerobic hydrocarbon impacted subsurface environment LCFA are generated from oxidative alkane activation reactions. Such activation reactions in isolation are energetically unfavourable. For instance, using the approach and assumed reaction conditions of Dolfing and colleagues (2008b) we calculated that methanogenic hexadecane degradation yields −371 kJ mol^−1^_(hexadecane)_. By contrast methanogenic degradation of hexadecanoate yields −385 kJ mol^−1^_(hexadecanoate)_. The difference between these two values (i.e. +14 kJ mol^−1^) represents the free energy cost for conversion of the alkane to the corresponding LCFA. The process is therefore endergonic and this investment of energy can only be recovered if the same organism is also able to utilize the LCFA generated. The coupling of alkane conversion to a fatty acid and LCFA oxidation by *Smithella* is certainly consistent with the first report of the methanogenic degradation of a pure alkane (hexadecane, Zengler *et al*., 1999) where three species from the *Syntrophaceae* were very highly enriched (90% of the bacterial population). These species shared 99%, 93% and 93% 16S rRNA sequence identity with the organisms enriched here on added crude oil alkanes (e.g. MO302D8, Fig. 4A, Table 3) and their almost exclusive enrichment in the degradation of hexadecane suggests their activation of this substrate with subsequent LCFA degradation.

### The role of *Marinobacter* in methanogenic oil degrading microcosms

Many *Marinobacter* spp. are aerobic marine heterotrophs, capable of growth on alkanes (e.g. Gauthier *et al*., 1992) and a number are known to degrade simple organic compounds with nitrate as an electron acceptor (Gauthier *et al*., 1992). Interestingly, *Marinobacter* spp. have been isolated from, and identified in, several anoxic hydrocarbon contaminated and subsurface environments (Huu *et al*., 1999; Orphan *et al*., 2000; Inagaki *et al*., 2003; Dunsmore *et al*., 2006; Gu *et al*., 2007, see Fig. 5). In these locations *Marinobacter* spp. are often considered non-indigenous aerobes or denitrifiers, although some deep subsurface isolates are considered indigenous (Batzke *et al*., 2007). A number of *Marinobacter* isolates, including some from subsurface marine sediments have been shown to be facultative anaerobes able to grow fermentatively (Köpke *et al*., 2005) and it was speculated that these organisms might be active *in situ* and participate in syntrophic interactions or use metal oxides as an electron sink (Köpke *et al*., 2005). This prompted us to evaluate the potential for anaerobic alkane degradation in the *Marinobacter* sp. that transiently increased in abundance in the methanogenic oil degrading microcosm clone library. The growth yield of *Marinobacter* (assuming that it was responsible for all alkane degradation) was only 0.0005 g_(cell-C)_/g_(alkane-C)_ thus it is unlikely that *Marinobacter* played an important role in methanogenic alkane degradation. These data are consistent with the lack of reports of *Marinobacter* spp. oxidizing alkanes under methanogenic conditions; however, some *Marinobacter* may be capable of the anaerobic oxidation of minor components of the crude oil in partnership with methanogens. Indeed the overall yield of methane in the microcosms was greater than can be explained by conversion of all the alkanes to methane and detailed analysis of the residual oil shows that compounds other than alkanes are removed, although the concentrations of these compounds are substantially lower than the alkane concentration. This suggests that additional organisms may be responsible for conversion of different components of crude oil to methane.

### Syntrophic partnerships in methanogenic crude oil degradation

In methanogenic environments organic carbon is degraded by syntrophic partnerships whereby participating organisms obtain energy by catalysing pathways that operate close to thermodynamic equilibrium (Dolfing *et al*., 2008b). For thermodynamic reasons bacterial syntrophy is sustainable only through the removal of the acetate, hydrogen or formate produced from fermentation of primary substrates and accordingly syntrophs rely on methanogens to consume these compounds (Dolfing *et al*., 2008b). MDS analysis of the microcosm communities showed a striking similarity between ordination patterns obtained with the archaeal and bacterial communities suggesting the establishment of such partnerships during methanogenic oil degradation.

In the oil amended microcosms, hydrogen-oxidizing *Methanomicrobiales* were enriched relative to facultative or obligate acetoclastic methanogens from the *Methanosarcinales*. Specifically, *Methanomicrobiales* sequences accounted for 82–94% of total methanogen growth and the calculated growth yield for this group [0.141–0.565 g_(cell-C)_/mol _(CH4)_] was broadly consistent but lower than values reported for pure cultures of methanogens belonging to the order *Methanomicrobiales*[0.64–1.47 g_(cell-C)_/mol _(CH4)_]. Members of the *Methanomicrobiales* lack cytochromes for energy conversion from hydrogenotrophic methanogenesis and, as such, have lower growth yields than members of the *Methanosarcinales* (Thauer *et al*., 2008).

Conventional wisdom suggests that the acetate generated from alkane oxidation should contribute two-thirds of methane production via acetoclastic methanogenesis (Table 2, Eq. 4). The predominance of hydrogen-oxidizing methanogens in subsurface environments (Head *et al*., 2010) may be explained by transport of additional hydrogen to petroleum reservoirs from external sources. These include hydrogen generated at high temperatures from organic matter maturation, serpentinization and radiolysis of water (Head *et al*., 2003). However, in our laboratory microcosms methanogens were only enriched in the presence of crude oil hydrocarbons and so there can be no external sources of hydrogen that would contribute to a predominance of hydrogenotrophic methanogens. An alternative explanation for hydrogenotroph enrichment in methangenic systems is SAO to H_2_ and CO_2_ (Table 2, Eq. 2, Zinder and Koch, 1984) coupled to methanogenesis from H_2_/CO_2_ (Table 2, Eq. 3). The possibility of SAO is supported in the present study by the formation of ^13^CO_2_ and a lower than predicted proportion of ^13^CH_4_ generated from ^13^CH_3_COO^-^ (Fig. 7), which indicates that both acetoclastic methanogens and syntrophic acetate oxidizers were active in the methanogenic oil degrading microcosms.

The contribution of SAO to acetate removal was affected by the initial acetate concentration. This finding is consistent with the balance of acetoclastic and non-acetoclastic methanogenesis in long-term acetate-fed chemostats inoculated with anaerobic digester sludge (Shigematsu *et al*., 2004). In a reactor with low acetate concentrations (10 mg l^−1^, 0.169 mM) 62–90% of methane was produced via SAO whereas in a reactor with high acetate concentrations (250 mg l^−1^, 4.2 mM) 95–99% of methane was derived from acetoclastic methanogenesis. In the methanogenic oil degrading microcosms, measured acetate concentrations were below detection limits (< 10 µM) throughout the incubation period consistent with the high proportion of SAO activity inferred from hydrogenotrophic methanogen growth yields. Propionic, isobutyric, butyric, isovaleric or valeric acids were also below detection limits.

In the methanogenic crude oil degrading systems studied here, enrichment of known syntrophic acetate oxidizers (e.g. *Clostridium* and *Thermoacetogenium* spp. Schnürer *et al*., 1996; Hattori *et al*., 2000; Hattori, 2008) was not observed. However, isotope tracer measurements and analysis of enrichment cultures suggest that SAO is a widely distributed phenotype in other phyla (e.g. Hori *et al*., 2007; Schwarz *et al*., 2007). For instance, in a stable isotope probing study using ^13^C-acetate enrichments of Florida Everglade eutrophic wetland soils (where SAO linked to hydrogenotrophic methanogenesis was the dominant methanogenic pathway) members of the *Syntrophaceae* were by far the most enriched bacteria (Chauhan and Ogram, 2006). On this basis the intriguing possibility exists that the *Smithella* sp. enriched in the oil degrading microcosms reported here and implicated in alkane oxidation via fatty acids was responsible for the complete degradation of alkanes to H_2_ and CO_2_ and ultimately to methane in syntrophic partnership with hydrogenotrophic methanogens.

## Experimental procedures

### Methanogenic hydrocarbon degrading microcosms

A description of the preparation of the methanogenic crude oil degrading microcosms is provided by Jones and colleagues (2008). Briefly, anaerobic microcosms were prepared with brackish carbonate buffered nutrient medium (100 ml) designed for the enrichment of sulfate reducing bacteria (Widdel and Bak, 1992) but without the addition of sulfate. Microcosms were inoculated with 10 g of sediment from the River Tyne, Newcastle, UK (54.96°N, 1.68°W). The methanogenic oil degradation experiments reported here comprised one experimental treatment and two control treatments [1. North Sea crude oil (300 mg); 2. North Sea crude oil (300 mg) plus 2-bromoethane sulfonate (BES; 10 mM final concentration) to inhibit methanogenesis; 3. No oil]. 18 replicate microcosms per treatment were prepared to allow for sacrificial sampling of triplicate microcosms at 22, 94, 176, 302, 450 and 686 days after inoculation. All microcosms were incubated in the dark in an anaerobic cabinet and routinely sampled for head space gas analysis (reported in Jones *et al*., 2008). Only microcosm samples from oil amended and unamended treatments were used for preparation of 16S rRNA gene clone libraries and qPCR analyses.

### Microbial community analyses

Before sacrificial sampling, microcosms were shaken to ensure homogeneity and vacuum filtered (10 ml) onto polycarbonate membrane filters (0.2 µm pore size, 13 mm diameter; Nucleopore, Whatman, Leicestershire, UK). DNA was extracted from the filters using a FastDNA Spin Kit for Soil (Q-BIOgene, California, USA), according to the manufacturer's instructions.

### PCR amplification of 16S rRNA gene fragments

Bacterial 16S rRNA gene fragments (∼ 1500 bp) were amplified by PCR using primers pA (5′-AGA GTT TGA TCC TGG CTC AG-3′) and pH reverse (5′-AAG GAG GTG ATC CAG CCG CA-3′) (Edwards *et al*., 1989). Archaeal 16S rRNA gene fragments (*c.* 1000 bp) were amplified with primers Arch46 (5′-YTA AGC CAT GCR AGT-3′) (Øvreås *et al*., 1997) and Arch1017 (5′-GGC CAT GCA CCW CCT CTC-3′) (Barns *et al*., 1994).

### DGGE analysis of PCR-amplified 16S rRNA gene fragments

Denaturing gradient gel electrophoresis analysis of bacterial 16S rRNA genes was conducted on DNA from microcosms sacrificially sampled on day 22, 302 and 686. Bacterial 16S rRNA gene fragments were subjected to a second round of amplification using primer 3 (5′-CCT ACG GGA GGC AGC AG-3′) containing a 5′ GC clamp and primer 2 (5′-ATT ACC GCG GCT GCT GG-3′) (Muyzer *et al*., 1993). Archaeal 16S rRNA gene fragments were subjected to a second round of amplification using Arch344 (5′-GAC GGG GHG CAG CAG GCG CGA-3′) containing a 5′ GC clamp (Raskin *et al*., 1994) and Uni 522 (5′-GWA TTA CCG CGG CKG CTG-3′) (Amann *et al*., 1995). PCR products were purified using a Qiagen PCR clean up kit (Qiagen, Crawley, UK). DGGE analysis was conducted using a D-Gene denaturing gradient gel electrophoresis system (Bio-Rad, Hercules, CA, USA) as previously described (Gray *et al*., 2002). Stained gels were viewed using a Fluor-S MultiImager (Bio-Rad, Hercules, CA, USA). The Bionumerics software package (Applied Maths, Austin, Texas, USA) was used to produce normalized composite gels with reference to marker lanes (van Verseveld and Röling, 2004), and band identity and relative intensity were determined for individual community profiles. Band matching data from this analysis were used to calculate Dice similarity indices for pairwise combinations of DGGE profiles. Dice similarities were used to calculate average similarity values of DGGE profiles from replicate microcosms and between treatments (oil amended and unamended). The mean similarities of community profiles were compared using Student's *t*-test.

### Cloning and sequencing of PCR-amplified 16S rRNA gene fragments

16S rRNA gene clone libraries were generated from the Tyne sediment used as an inoculum and from single replicate microcosms sampled from each treatment at 22, 302 and 686 days. Single representative samples were used on the basis of high similarity of DGGE profiles from replicate microcosms subject to the same treatments (*c.* 70–85% similarity; Table 1). PCR-amplified 16S rRNA gene fragments were cloned using a TOPO TA Cloning kit (Invitrogen, Paisley, UK) using the pCR4-TOPO vector according to the manufacturer's instructions. Randomly selected clones were screened to determine insert size using PCR with the vector-specific primers pUCF. (5′-GTT TTC CCA GTC ACG AC-3′) and pUCR (5′-CAG GAA ACA GCT ATG AC-3′). Cloned inserts of the correct size in PCR reactions (5 µl) were purified using ExoSAP-IT (2 µl; GE Healthcare, Buckinghamshire, UK), according to the manufacturer's instructions. Sequencing was performed on an ABI Prism 3730xl DNA sequencer (Applied Biosystems, Warrington, UK) by the Institute for Research on Environment and Sustainability (IRES) sequencing service. (Newcastle University, UK). Sequences were compared to the EMBL Nucleotide Sequence Database at the European Bioinformatics Institute using Fasta3 (Pearson and Lipman, 1988) to identify the nearest neighbours. Initially, ∼ 500 nucleotides of sequence read was obtained from the primer pC (Edwards *et al*., 1989) for bacterial clones and the primer Arch 46 (Øvreås *et al*., 1997) for archaeal clones. Sequence quality was determined using Chromas 2.3 (Technelysium Pty; http://www.technelysium.com.au/chromas.html). Sequences were assigned to the Ribosomal Database Project (RDP release 10) taxonomic hierarchy using the online classifier tool (Wang *et al*., 2007). In addition, sequences were imported into the tree-building and database management software ARB, aligned and inserted into reference bacterial or archaeal trees in ARB using the quick parsimony insertion tool (Ludwig *et al*., 2004). The resultant trees were used to further refine taxonomic classifications. For the day 302 methanogenic oil degrading bacterial clone library phylogenetic reconstruction was refined by obtaining longer sequences (approximately 900 bp) for OTUs sharing less than 99% sequence identity. These longer sequences were obtained using additional primers T3 (5′-AATTAACCCTCACTAAAGGGA-3′) or T7 (5′-GTAATACGACTCACTATAGGGC-3′). Longer archaeal sequences (approximately 1000 bp) were obtained by using the primer Arch 1017 (Barns *et al*., 1994). Assembly of sequencing reads was performed in BioEdit (Hall, 1999) using the Contig Assembly Program (CAP; Huang, 1992). The presence of chimeric sequences within the data set were determined using Mallard (Ashelford *et al*., 2006) and/or Pintail (Ashelford *et al*., 2005) available from http://www.bioinformatics-toolkit.org. All 16S rRNA sequences have been deposited in the GenBank database with accession numbers (GU996298-GU997026). Neighbour joining distance trees for the longer sequences were constructed with reference sequences from GenBank selected to represent cultured and uncultured close relatives. Trees were constructed using the method of Saitou and Nei (Saitou and Nei, 1987) with the Jukes and Cantor correction for multiple substitutions at a single site (Jukes and Cantor, 1969). Bootstrap re-sampling was conducted with 100 replicates using the TREECON package (van De Peer and De Wachter, 1994).

### Analysis of bacterial and archaeal community composition

Comparison of 16S rRNA gene clone libraries was based on rank abundance data for different OTUs identified. The rank abundance data were analysed by non-metric MDS (Clarke and Warwick, 2001) using Primer 6 community analysis software (PRIMER 6 for Windows; Version 6.1.5, PRIMER -E Ltd, UK). To determine which OTUs most influenced clustering of the data the contribution of each OTU was disaggregated using the SIMPER routine in PRIMER whereby species were ordered by their average contribution to the dissimilarities of the communities in the oil-treated and unamended microcosms. In addition, pairwise comparisons of clone libraries were made using the RDP Naive Bayesian rRNA Classifier Version 2.2, which provides estimates of the significance of differences in a given taxon between clone libraries.

### Quantification of 16S rRNA genes in methanogenic oil degrading microcosms

The abundance and dynamics of specific bacterial and archaeal groups within the oil amended and unamended microcosms were determined by quantitative real time PCR (qPCR). The choice of primer pairs was based on the identity of OTUs, which were enriched or depleted in the oil amended microcosms (Table 4). For instance, a primer pair targeting a subgroup of the family *Syntrophaceae* within the *Deltaproteobacteria* (specifically, the genera *Smithella* and *Syntrophus*) was designed for this study along with a primer pair targeting a group of sequences from the genus *Marinobacter* (*Gammaproteobacteria*), which included *Marinobacter hydrocarbonoclasticus* strain VT8 (AJ000726) and sequences identified in the oil amended microcosms. The design of these primer pairs was accomplished using the probe and PCR primer design software tool Primrose (Ashelford *et al*., 2002) incorporating full-length sequences for the target groups (i.e. 15 *Smithella* and *Syntrophus* sequences and 5 *Marinobacter* affiliated clone sequences). Candidate primers were then screened against a larger database constructed within PRIMROSE, which included (in addition to the RDP release 8 database) sequences from all *bona fide Syntrophacaeae* and *Alteromonadales incertae sedis* 7 present in RDP release 10. Candidate primer sequences were screened (see Table 4) for specificity using the RDP probe match analysis tool (Cole *et al*., 2007). In addition, the specificity of a primer pair targeting most bacteria, adapted from Maeda *et al*. (2003), was tested along with three primer pairs (Yu *et al*., 2005) targeting different archaeal groups (*Methanomicrobiales, Methanosarcinaceae* and *Methanosaetaceae*). Quantification was performed on DNA extracts from all of the oil amended and unamended microcosms for all of the sacrificial sampling time points (22, 94, 176, 302, 450 and 686 days). Gene abundance in microcosm DNA samples was determined in relation to calibration standards from a 10-fold dilutions series (10^8^–10^1^ gene copies per µl) of target DNA sequence. The DNA targets were derived from clone sequences obtained from the oil degrading microcosms.

**Table 4:** Quantitative PCR primers used in this study

Target group	Primer	Sequence (5′ to 3′)	Size (bp)	Annealing temperature (°C)	Reference	Target group matches	Non-target matches
Bacteria	U1048f	GTG ITG CAI GGI IGT CGT CA	323	60.5	This study^a^	93%	0 sequences
	U1371	ACG TCI TCC ICI CCT TCC TC					
*Syntrophus* + *Smithella*	Syn 827f	TTC ACT AGG TGT TGR GRG	436	59.6	This study	88% & 77%^b^	0 sequences
	Syn 1263r	CTC TTT GTR CCR CCC ATT					
*Marinobacter*	Mab 451f	TGG CTA ATA CCC ATG AGG	122	60	This study	9%^b^,^c^	2 sequences^c^
	Mab 573r	TAG GTG GTT TGG TAA GCG					
*Methanomicrobiales*	MMB 282f	ATC GRT ACG GGT TGT GGG	550	66	Yu *et al*. (2005)	90%	0.4%^b^,^d^
	MMB 832r	CAC CTA ACG CRC ATH GTT TAC					
*Methanosarcinaceae*	Msc 380f	GAA ACC GYG ATA AGG GGA	448	62	Yu *et al*. (2005)	45%	0.7%^b^,^e^
	Msc 828r	TAG CGA RCA TCG TTT ACG					
*Methanosaetaceae*	Mst 702f	TAA TCC TYG ARG GAC CAC CA	126	62	Yu *et al*. (2005)	71%	0.2%^b^,^e^
	Mst 826r	CCT ACG GCA CCR ACM AC					

a Adapted from Maeda and colleagues (2003).

b Primer pair targets all target group sequences found in the day 302 and 686 clone libraries but no non-target sequences.

c The *Marinobacter* primer pair targeted only a small subgroup (9.2%) of the *Marinobacter* genus including *Marinobacter hydrocarbonoclasticus* (T); VT8; AJ000726. The forward and reverse primers shared two non-target sequences namely, the uncultured bacterium; F20; AY375115 currently assigned by RDP to the alphaproteobacterial genus *Erythrobacter* and an unclassified proteobacterium ctg_NISA064; DQ396144.

d Indicates only non-target matches within the class *Methanosarcinales.*

e Indicates only non-target matches within in the class *Methanomicrobiales*. The *Methanosarcinaceae* primers targeted approximately 12% of *Methanosaetaceae* and the *Methanosaetaceae* primers targeted approximately 0.3% of the *Methanosarcinaceae.*

I, inosine.

qPCR reactions comprised iQ Supermix (10 µl), PCR primers (1 µl of 10 pmoles µl^−1^ each), sterile water (6 µl), SYBR Green (0.2 µl per reaction of 100 × diluted from 10 000 × concentrate) and DNA template (3 µl) made up to a final volume of 20 µl. qPCR reactions were carried out using a Bio-Rad iQ5 thermocycler and included an initial denaturation (7 min at 95°C), followed by 40 cycles (for the bacterial primer pair) or 55 cycles (for all other primer pairs) of [30 s at 95°C, 30 s at the specific primer annealing temp (see Table 3) and 40 s at 72°C]. Optimal annealing temperatures were determined for the *Syntrophaceae* and *Marinobacter* primer pairs by performing a temperature gradient PCR with annealing temperatures in the range of 57°C to 70°C. Target 16S rRNA gene templates were PCR-amplified from bacterial and archaeal clones obtained and sequenced in this study. The target clone sequences were amplified using primers pUCf and pUCr as described above. The DNA concentrations were measured spectrophotometrically using a NanoDrop ND-1000 spectrophotometer. To improve PCR efficiencies standard dilution series containing known concentrations of target 16S rRNA gene fragments were prepared by mixing the highest concentration standard (10^9^ genes µl^−1^) with a complex mixture of PCR-amplified bacterial 16S rRNA genes obtained from River Tyne sediment DNA. In the presence of this background matrix all the target standard dilution series gave high correlation coefficients (above 0.99), similar calibration slopes (between −3.0 and −3.9) and qPCR efficiencies (> 80%). Without adding this background matrix the efficiency of some of the qPCR assays determined with pure standard was consistently low. The background contribution of *Syntrophus/Smithella* and Marinobacter genes present in River Tyne sediment derived 16S rRNA matrix used to prepare the standard dilution series was estimated at less than 1% of the respective added amounts of standard target DNA.

### Microcosm cell size estimates

Microcosm samples (0.5 ml) were fixed by addition of 0.5 ml molecular biology grade filtered absolute ethanol (0.2 micron filtered) and stored at −20°C. SYBR-gold nucleic acid stain (Invitrogen, Paisley, UK) (50 µl diluted 100-fold) was added to fixed samples (1 ml) and incubated in the dark at room temperature for 30 min. After incubation cells were vacuum filtered onto polycarbonate membrane filters (Isopore, 13 mm, 0.2 µm pore size, Millipore, Watford, UK), washed in 1 × phosphate-buffered saline (130 mM NaCl, 10 mM sodium phosphate, pH 7.4), and covered with a cover slip on a standard microscope slide. Cells were viewed under oil immersion (100 ×) on an epifluorescence microscope (BX40, Olympus, London, UK), under a blue light filter. Images were captured using a digital camera (Olympus E-400, Olympus, UK) and cell size estimates were obtained using the Cell C image analysis software (Selinummi *et al*., 2005, https://sites.google.com/site/cellcsoftware/).

### Growth characteristics derived from qPCR data

Specific growth rates *µ* were calculated from qPCR derived cell abundance using ln *N_T_* − ln *N_0_* = *µ t* where *N_0_* is the number of cells at the start of the exponential growth phase, *N_T_* is the number of cells at time *T*, and *t* is the time elapsed in days. Doubling times were calculated as ln2/µ). Growth yields (g _cell carbon_/g _carbon from alkane_) were calculated using alkane removal data from Jones and colleagues (2008) and biomass carbon. Biomass carbon was estimated from qPCR derived gene abundances as follows. Cell numbers were derived from qPCR data by dividing 16S rRNA gene abundances by the rRNA operon copy number for the different taxa analysed (*Smithella/Syntrophus* 1 copy, *Marinobacter* 3 copies, *Methanomicrobiales* 1–4 copies, *Methanosarcinaceae* 3 copies and *Methanosaetaceae* 3 copies) obtained from the ribosomal RNA operon copy number database (rrnDB) (Lee *et al*., 2009). Cell numbers were converted to cell volumes using the average measured cell volume (0.024 µm^3^) determined from the hydrocarbon degrading microcosm samples. Finally, cell volumes were converted to carbon content based on an assumption of 310 fg C µm^−3^ (Fry, 1990).

### Measurement of SAO

Syntrophic acetate oxidation in samples from the methanogenic crude oil degrading microcosms was measured in glass serum bottles (14 ml, Aldrich, UK) sealed with butyl rubber stoppers and aluminium crimps (Aldrich, UK). Replicate incubations were prepared with anaerobic carbonate buffered nutrient medium (5 ml; Widdel and Bak, 1992) and different amounts of 2-^13^C sodium acetate, 99 Atom%, Sigma Aldrich UK). The final concentration of added labelled acetate in each treatment was 10, 1, 0.1 or 0 mM). The serum bottles were inoculated with 1 ml from a methanogenic oil degrading microcosm, which had been incubated for 450 days. All microcosms were incubated on a shaker (100 r.p.m.) at 22°C. Headspace gases were periodically analysed for ^13^C- and ^12^C-labelled CH_4_ and CO_2_ (Gray *et al*., 2006).

## Supporting information

**Fig. S1.** Bacterial and archaeal DGGE profiles from replicate oil amended and unamended microcosms sacrificed on day 302.

**Fig. S2.** Phylogenetic affiliation of non-*Proteobacterial* 16S rRNA sequences recovered from methanogenic microcosms. Clone frequency in bacterial 16S rRNA gene clone libraries from the inoculum and initial day 22 samples (bottom panel, total number of clones = 71) and in samples from methanogenic oil degrading microcosms (top panel, number of ‘day 302’ clones = 61, number of ‘day 686’ clones = 87) and control microcosms with no added oil (middle panel, number of ‘day 302’ clones = 62, number of ‘day 686’ clones = 87). Data from clone libraries from day 302 (filled bars) and day 686 (open bars) are shown. Clones were grouped into categories based on their genus, order, class or phylum level affiliation after phylogenetic analysis with the ARB software package using an RDP guide tree. The affiliation of individual sequences was cross-checked using the RDP taxonomical hierarchy with the Naive Bayesian rRNA Classifier Version 2.0, July 2007. DGGE profiles from replicate microcosms were highly reproducible and clone libraries were prepared for one representative replicate microcosm for each time point.

**Fig. S3.** Phylogenetic distance trees based on comparative analysis of non-*Proteobacterial* partial 16S rRNA sequences recovered from a representative oil amended microcosm on day 302. Sequences recovered in this study (grey text) are prefixed by MO302. Related organisms identified in petroleum systems or those directly implicated in oil degradation are in bold. GenBank accession numbers for all database sequences are provided in parenthesis. Tree rooted with respect to the *Marinobacter excellens* KMM 3809^T^ 16S rRNA sequence (AY180101). The scale bar denotes 10% sequence divergence and the values at the nodes indicate the percentage of bootstrap trees that contained the cluster to the right of the node. Bootstrap values less than 50 are not shown.
