# Supplementary figures and images for: The quantitative significance of Syntrophaceae and syntrophic partnerships in methanogenic degradation of crude oil alkanes
Source: Environ Microbiol. 2011 Nov;13(11):2957–75. doi: 10.1111/j.1462-2920.2011.02570.x (PMC3258425; doi:10.1111/j.1462-2920.2011.02570.x)

Gray et al. Figure S1

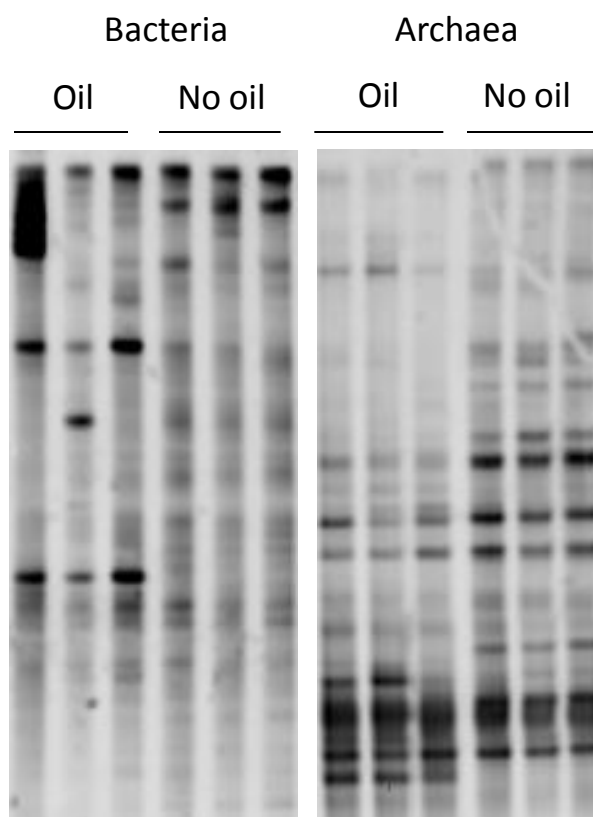

Supplement: Supplementary file 1 [file emi0013-2957-SD1.pdf]

# Gray et al. supplementary Figure S2

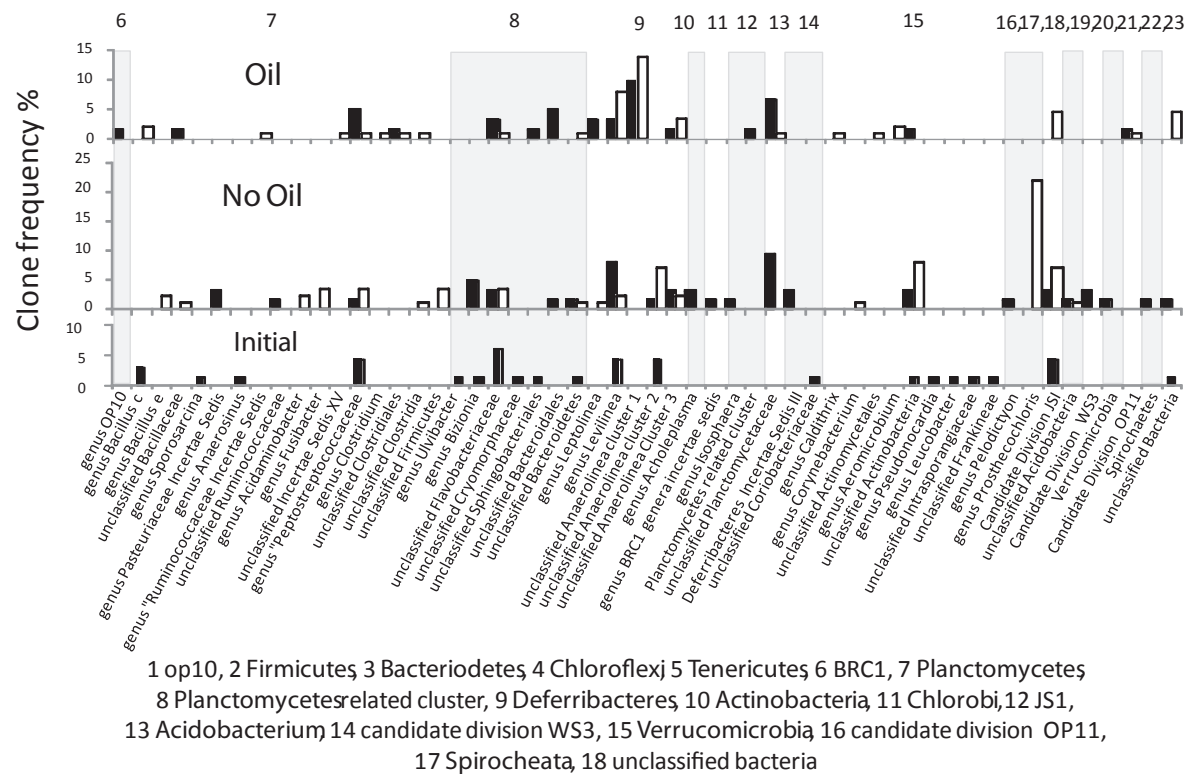

Supplement: Supplementary file 2 [file emi0013-2957-SD2.pdf]

# Gray et al. supplementary Figure S3

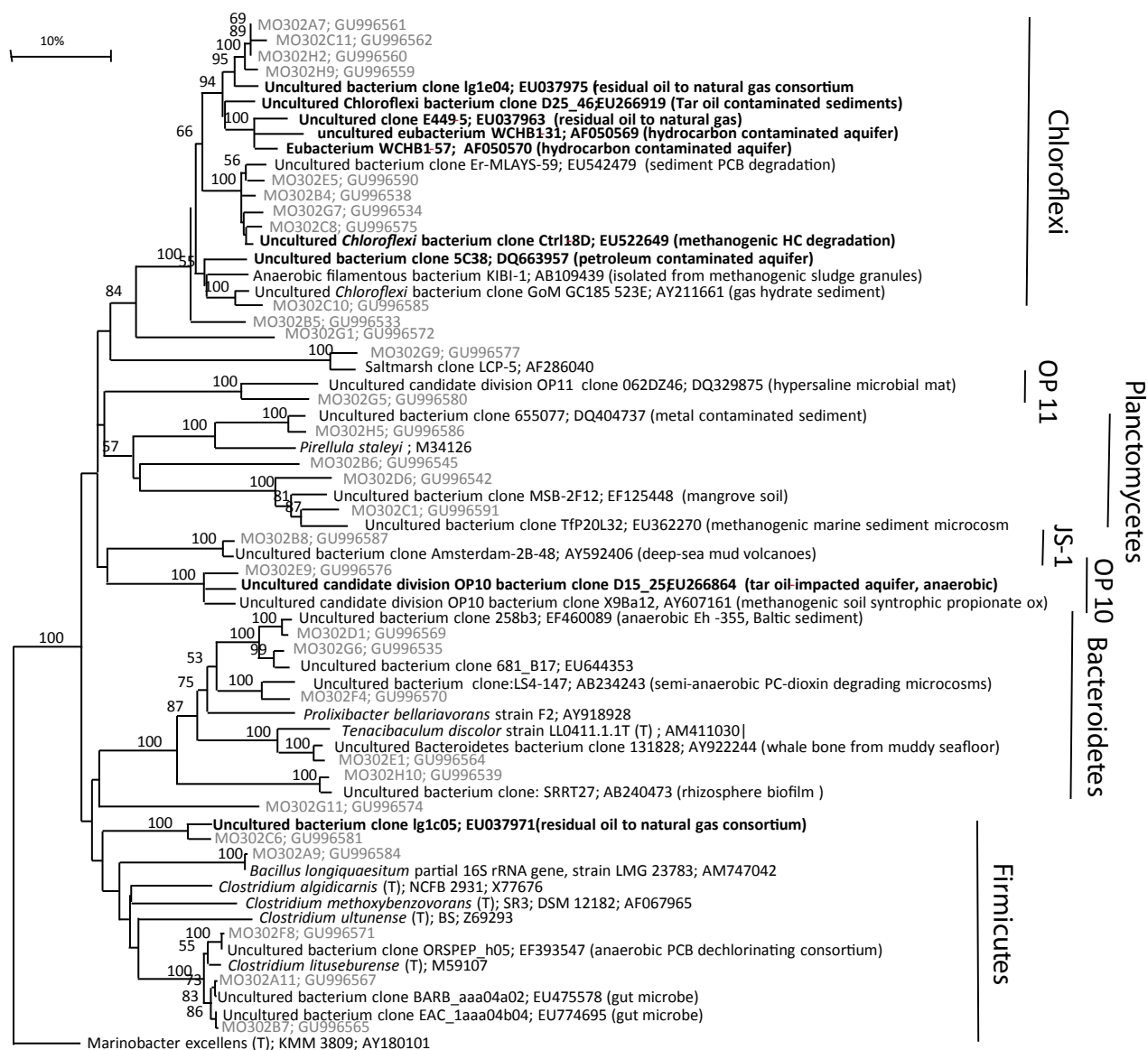

Supplement: Supplementary file 3 [file emi0013-2957-SD3.pdf]
