# Supplementary material for: The quantitative significance of Syntrophaceae and syntrophic partnerships in methanogenic degradation of crude oil alkanes
Source: Environ Microbiol. 2011 Nov;13(11):2957–75. doi: 10.1111/j.1462-2920.2011.02570.x (PMC3258425; doi:10.1111/j.1462-2920.2011.02570.x)
Supplement: Supplementary file 4 [file emi0013-2957-SD4.doc]

**Supplementary figure S1.** Bacterial and archaeal DGGE profiles from replicate oil amended and unamended microcosms sacrificed on day 302.

**Supplementary figure S2.** Phylogenetic affiliation of non-*Proteobacterial* 16S rRNA sequences recovered from methanogenic microcosms. Clone frequency in bacterial 16S rRNA gene clone libraries from the inoculum and initial day 22 samples (bottom panel, total number of clones = 71) and in samples from methanogenic oil degrading microcosms (top panel, number of ‘day 302’ clones = 61, number of ‘day 686’ clones = 87) and control microcosms with no added oil (middle panel, number of ‘day 302’ clones = 62, number of ‘day 686’ clones = 87). Data from clone libraries from day 302, (filled bars) and day 686 (open bars) are shown. Clones were grouped into categories based on their genus, order, class or phylum level affiliation after phylogenetic analysis with the ARB software package using an RDP guide tree. The affiliation of individual sequences was crosschecked using the RDP taxonomical hierarchy with the Naive Bayesian rRNA Classifier Version 2.0, July 2007. DGGE profiles from replicate microcosms were highly reproducible and clone libraries were prepared for one representative replicate microcosm for each time point.

**Supplementary figure S3.** Phylogenetic distance trees based on comparative analysis of non-*Proteobacterial* partial 16S rRNA sequences recovered from a representative oil amended microcosm on day 302. Sequences recovered in this study (grey text) are prefixed by MO302. Related organisms identified in petroleum systems or those directly implicated in oil degradation are in bold. Genbank accession numbers for all database sequences are provided in parenthesis. Tree rooted with respect to the *Marinobacter excellens* KMM 3809^T^ 16S rRNA sequence (AY180101). The scale bar denotes 10% sequence divergence and the values at the nodes indicate the percentage of bootstrap trees that contained the cluster to the right of the node. Bootstrap values less than 50 are not shown.
